# Tenecteplase versus alteplase in bridging therapy in patients with large vessel occlusion stroke: a meta-analysis

**DOI:** 10.3389/fneur.2025.1661357

**Published:** 2025-11-05

**Authors:** Xiaohan Zhang, Min Tao, Tian Wang, Rongxin He, Lingwan Yan, Xiaozuo Lin, Pingkai Wang, Yinan Zeng, Jipeng Yu, Hongxia Liu, Man Luo

**Affiliations:** Department of Neurology, The First Affiliated Hospital of Guangxi Medical University, Nanning, Guangxi, China

**Keywords:** acute ischemic stroke, tenecteplase, alteplase, intravenous thrombolysis, mechanical thrombectomy

## Abstract

**Background:**

Currently, for patients with large-vessel occlusion (LVO) strokes, the standard treatment approach involves using alteplase (ALT) as a bridge to endovascular mechanical thrombectomy (MT). Tenecteplase (TNK) is a novel fibrinolytic agent. Our research is focused on evaluating and comparing the efficacy and safety of TNK and ALT in patients with acute ischemic stroke (AIS) and large-vessel occlusion before they undergo MT.

**Methods:**

The study’s research plan was registered in the International Prospective Register of Systematic Reviews (PROSPERO) under the number CRD42025643339. The entire process adhered to the Preferred Reporting Items for Systematic Reviews and Meta-Analyses (PRISMA) 2020 guidelines, guaranteeing high-quality and standardized reporting and analysis.

**Results:**

In total, 7 studies involving 4,580 patients were incorporated. Patients treated with TNK exhibited comparable rates of functional independence at 90 days (odds ratio 1.23, 95% confidence interval 0.90–1.68, *p* = 0.2), post-MT recanalization (1.18, 0.93–1.51, *p* = 0.18), symptomatic intracerebral hemorrhage (sICH; 1.01, 0.62–1.65, *p* = 0.98) and mortality within 90 days (0.77, 0.51–1.18, *p* = 0.24) to those treated with alteplase. However, compared to alteplase-treated patients, those treated with TNK had higher rates of early recanalization (1.28, 1.06–1.53, *p* = 0.009), and a lower incidence of intracranial hemorrhage (ICH; 1.83, 1.26–2.66, *p* = 0.002).

**Conclusion:**

Regarding of functional independence at 90 days, post-MT recanalization, sICH and 90-day mortality in AIS patients undergoing MT, there were essentially no difference between TNK and ALT. However, TNK might be more effective than ALT in achieving early recanalization, and it may also reduce the risks of ICH.

**Clinical trial registration:**

Unique Identifier: CRD42025643339, Publicly Accessible URL: https://www.crd.york.ac.uk/PROSPERO/.

## 1 Introduction

Worldwide, AIS ranks among the primary causes of both mortality and disability (1, 2). MT serves as an effective treatment modality for LVO strokes. In recent years, numerous trials have validated its safety and effectiveness in treating such strokes, with its applicability extending up to 24 h from the onset of the stroke. MT has had a significant impact in reducing mortality rates. Nevertheless, despite achieving high recanalization rates, a large number of clinical outcomes still demonstrate less-than-ideal functional recoveries (3–8). In the following randomized controlled trials (RCTs), a comparison was made between direct MT, where intravenous thrombolysis (IVT) was not administered, and the approach of using IVT as a bridging therapy in conjunction with MT for patients suffering from acute vessel occlusion (9–12). Significantly, within these particular RCTs, ALT was the only IVT agent predominantly employed. At present, for patients with AIS within 4.5 h after stroke onset, ALT is the most frequently utilized thrombolytic drug recommended by guidelines (13). Currently, MT is the core treatment method for LVO strokes, and the bridging strategy combining IVT with MT is the standard approach recommended by the guidelines. Among them, ALT is the traditional IVT drug, but it has limitations such as a short half-life, low early recanalization rate, and the risk of ICH (14–16). TNK, as a new generation of recombinant tissue plasminogen activator (rtPA), has the advantages of high fibrin specificity and single-dose administration, and has shown better reperfusion potential in some studies (17–21). However, the efficacy and safety differences between TNK and ALT in bridging treatment remain controversial. Recent key trials have provided important evidence in this field: The BRIDGE-TNK trial as a landmark study first confirmed that the bridging scheme of intravenous TNK combined with MT is superior to direct MT, significantly improving functional independence at 90 days (22); The EXTEND-IA TNK trial showed that TNK in LVO patients had a higher early recanalization rate and better 90-day functional prognosis than ALT (23); The TIMELESS trial suggested that the efficacy of TNK combined with MT within the 4.5–24 h window is comparable to that of MT alone (24). The AcT trial found that there was no significant difference in recanalization rate and 90-day functional prognosis between TNK and ALT in the AIS patients (25). The inconsistencies in these research results, especially the real impact of TNK on early recanalization and ICH risk in bridging treatment, have not yet been clearly answered by integrating the latest evidence. Moreover, most existing meta-analyses do not include the latest relevant studies and do not focus on the bridging treatment scenario for LVO patients. Therefore, this study systematically compares the efficacy and safety of TNK and ALT as pre-bridging treatments before MT by including recent RCTs and high-quality observational studies, aiming to fill the current evidence gap and provide more reliable evidence for clinical decision-making.

Although existing meta-analyses comparing TNK and ALT have confirmed the superiority of TNK in terms of early recanalization rate (26–28), they still have three limitations: First, some studies included non-LVO populations included acute ischemic stroke patients without large vessel occlusion (27), which may dilute the effect size in the context of bridging therapy. Second, most studies did not incorporate key cohort studies published after 2023 or the latest RCTs in 2025, thus failing to reflect recent clinical practice data. Third, no previous study has focused on “ICH risk stratification in bridging therapy”; instead, they only conducted an overall comparison of sICH and failed to reveal the unique value of TNK in reducing asymptomatic ICH. By strictly restricting the study population to patients with LVO undergoing MT, including high-quality studies published in the past 3 years, and quantifying the effect of TNK on reducing ICH incidence for the first time (1.83, 1.26–2.66, *p* = 0.002), this study fills the gap in the existing evidence.

## 2 Methods

### 2.1 Study protocol

The entire research process was executed in strict compliance with the PRISMA 2020 guidelines (29) (Supplementary File 1). And the study protocol has been registered in the International PROSPERO with the unique identifier CRD42025643339. Considering the specific nature of this analysis, it was determined that approval from the local Institutional Review Board was not required.

### 2.2 Search strategy

Two experienced reviewers carried out a systematic search across four major databases: PubMed, Embase, Web of Science, and the Cochrane Library to identify all relevant studies, commencing from the earliest records available in these databases up to August 25, 2025. Search strategy involved a combination of specific terms, including “Alteplase,” “Tenecteplase,” “Ischemic Stroke,” “Ischaemic Stroke,” “Cerebral Infarction,” “Middle Cerebral Artery Stroke,” “Middle Cerebral Artery Infarction,” and “Thrombectomy.” These terms were utilized as both keywords and Medical Subject Headings (MeSH) terms (Supplementary File 2). To identify any potentially overlooked eligible studies, we meticulously examined the reference lists of the trials we had included. This was done because these might contain additional relevant research that wasn’t detected during the initial screening of databases.

### 2.3 Selection criteria

This review encompassed cohort studies (both prospective and retrospective) and randomized controlled trials. These studies focused on adult (≥18-year-old) patients with AIS who received bridging therapy using either TNK or ALT prior to undergoing MT. We restricted the scope of the included studies to those published in the English language. we will specify that the included studies must be “high-quality RCTs (defined by the Cochrane Risk of Bias tool)” or “observational studies with a cohort design that meets the Newcastle-Ottawa Scale (NOS) ≥ 7 stars.” Additionally, we excluded case reports, small-sized case series (with fewer than 20 cases), conference proceedings, and review articles. Only studies that conducted a comparative analysis between TNK and ALT were incorporated. Studies that reported findings of only one of the agents or did not report the use of MT were excluded. When duplicate authors and/or participating centers were found among several studies, we chose only the study having the largest sample size for our analysis.

### 2.4 Data extraction

Two authors (Xiaohan Zhang and Min Tao) conducted a comprehensive review of all seven studies. From each of the final-selected studies, two reviewers independently extracted data on the quantity of patients and the outcomes of concern in the TNK and ALT groups before they underwent MT, including year, country, study design, the quantity of patients in total, the dosage of thrombolytic agent, the occluded site, age, sex, median National Institute of Health stroke scale (NIHSS) score, outcomes measure (Table 1). In the course of this data-retrieval procedure, in case any differences emerged, they were settled through dialogue and agreement after conferring with a third reviewer (Tian Wang). The main outcome measure for this study was functional independence at 90 days, which was defined as having a modified Rankin Scale (mRS) score between 0 and 2. In terms of safety, the indicators considered were sICH and ICH. ICH was characterized as any kind of intracerebral bleeding that could be detected through imaging methods or measured by a hemorrhage-related scoring system. sICH was defined as ICH that was temporally related to and directly contributing to the deterioration of the neurological condition. sICH: According to the European Cooperative Acute Stroke Study III (ECASS III) standard (Imaging confirmed ICH with an increase of≥4 points in the NIHSS score), and the Safe Implementation of Thrombolysis in Stroke-Monitoring Study (SITS-MOST) standard (sICH occurs within 36 h after IVT, and neurological deterioration is directly related to sICH). The specific definitions of sICH adopted in each included study are shown in Table 2 (30). Regarding the secondary outcomes, successful recanalization after MT was one of them. This was determined by a post-MT modified Thrombolysis in Cerebral Ischemia (mTICI) score of 2b/3, which indicated that more than 50% of the ischemic area had been reperfused (31). Another secondary outcome was the rate of early successful recanalization. This was defined as the first angiographic assessment reaching mTICI 2b/3 grade. It should be noted that some patients who achieved mTICI 2b grade may still undergo MT due to a high residual thrombus burden or insufficient perfusion. This definition may introduce potential classification bias, and the related effects have been discussed in the subsequent sections. Also included as a secondary outcome was all-cause mortality within 90 days.

**Table 1:** Characteristics of included studies.

Study	Year	Country	Study design	No. Patients (TNK/ALT)	Dose(s) mg/kg (TNK/ALT)	Occluded site	Age, in years [mean ± SD or median (IQR)]	Sex, *n* (Male/Female)	Media NIHSS score (IQR)	Outcome measure
Campbell (23)	2018	Australia and New Zealand	RCT	101/101	0.25/0.9	Anterior+posterior circulation	71.15 ± 14.44	110/92	TNK:17 (12–22) ALT:17 (12–22)	ER, Post-MT recanalization, sICH, ICH, FI at 90 days, Mortality within 90 days
Checkouri (34)	2023	France	Prospective observational study	787/1078	0.25/0.9	Anterior circulation	70.2 ± 15.27	917/948	TNK:16 (10–20) ALT:16 (11–20)	ER
Hendrix (36)	2024	American	Retrospective comparative study	309/326	0.25/0.9	Anterior+posterior circulation	69.14 ± 14.23	320/315	TNK:17 (11–22) ALT:17 (12–22)	ER, Post-MT recanalization, sICH, FI at 90 days, Mortality within 90 days
Marnat (37)	2023	France	Prospective observational study	124/629	0.25/0.9	Anterior circulation	65.13 ± 13.68	536/217	TNK:15 (9–19) ALT:16 (11–20)	ER, Post-MT recanalization, sICH, ICH, FI at 90 days, Mortality within 90 days
Teivane (35)	2022	Latvia	Retrospective comparative study	45/139	0.25/0.9	Anterior+posterior circulation	71.88 ± 12.07	112/72	TNK:14 (4–26) ALT:15 (2–31)	ER, Post-MT recanalization, ICH, FI at 90 days, Mortality within 90 days
Alhabli (38)	2025	Canada	RCT	252/244	0.25/0.9	Anterior+posterior circulation	-	252/244	TNK:17 (11–22) ALT:17 (9–20)	ER
Diprose (39)	2025	Canada	Retrospective comparative study	226/219	0.25/0.9	Anterior+posterior circulation	73 (63–81)	215/230	-	ER, Post-MT recanalization

SD, standard deviation; IQR, interquartile range; NIHSS, National Institutes of Health Stroke Scale; RCT, randomized controlled trial; TNK, tenecteplase; ALT, alteplase; ER, early recanalization; MT, mechanical thrombectomy; FI, functional independence; ICH, intracerebral hemorrhage; sICH, symptomatic intracerebral hemorrhage.

**Table 2:** Definition of sICH.

First author (year published)	sICH criteria	Judge reference
Campbell (2018) (23)	SITS-MOST	Imaging and clinical deterioration within 36 h
Hendrix (2024) (36)	SITS-MOST	Imaging and clinical deterioration within 36 h
Marnat (2023) (37)	ECASS-III	NIHSS changes and CT/MRI

sICH, Symptomatic intracranial hemorrhage; SITS-MOST, Safe Implementation of Thrombolysis in Stroke-Monitoring Study; ECASS, The European Cooperative Acute Stroke Study; CT, Computed Tomography; MRI, Magnetic Resonance Imaging.

### 2.5 Risk of bias assessment

The Cochrane risk of bias tool was used to evaluate 2 randomized controlled trials, and the Newcastle-Ottawa Scale (NOS) was employed to assess the risk of bias for 5 cohort studies. We used the risk of bias tool to assess bias, covering six domains: selection bias, performance bias, detection bias, attrition bias, reporting bias and other bias (32). The Newcastle-Ottawa Scale (NOS) was employed by us to evaluate the risk of bias in cohort studies as well as post-hoc analyses of RCTs (33). The NOS assesses the quality of a study across three main aspects: the selection of study participants, the comparability among different study groups, and the assessment of study outcomes. A study scoring 3 stars or less, 4 to 6 stars, or 7 to 9 stars is, respectively, regarded as being of low quality, moderate quality, or high quality. Two evaluators (Xiaohan Zhang and Min Tao) independently conducted bias risk assessment using the Cochrane RoB tool (for RCTs) and the NOS scale (for cohort studies). In case of disagreement, they reached a consensus through a blind discussion with the third evaluator (Tian Wang). The risk of publication bias between studies was assessed using funnel plot symmetry and egger test.

### 2.6 Statistical analysis

In this meta-analysis, we made use of random-effects models to analyze the outcome data. For the primary analyses, we computed the odds ratios (ORs) and their corresponding 95% confidence intervals (CI) for each outcome under consideration. We adopted the Mantel–Haenszel method within the framework of random-effects models for these calculations. This study clearly defined the following subgroup analysis content in advance: based on the location of the occlusion, TNK dose and experimental design. The subgroup analysis aimed to explore the sources of heterogeneity and the analysis method was consistent with the main analysis. In the sensitivity analysis, the random effects model was used to weight observational studies of different qualities. The weights were comprehensively considered based on the sample size of the study (the larger the sample size, the higher the weight) and the NOS score (the weight of high-quality studies (7–9 stars) was higher than that of medium-quality studies), in order to reduce the interference of low-quality studies on the combined effect. We utilized the *χ^2^* test and the *I^2^* statistic to assess the statistical heterogeneity within the incorporated studies. A determination of substantial heterogeneity was made when the value of the *I*^2^ statistic surpassed 50%. The Egger test using Stata 17.0 software and the funnel plot using RevMan 5.4 software were employed to assess publication bias. Meta-analysis was conducted using RevMan 5.4 software. In this study, statistical significance was considered to be indicated when the two-tailed *p* value was less than 0.05.

## 3 Results

### 3.1 Literature retrieval

**Figure 1:**
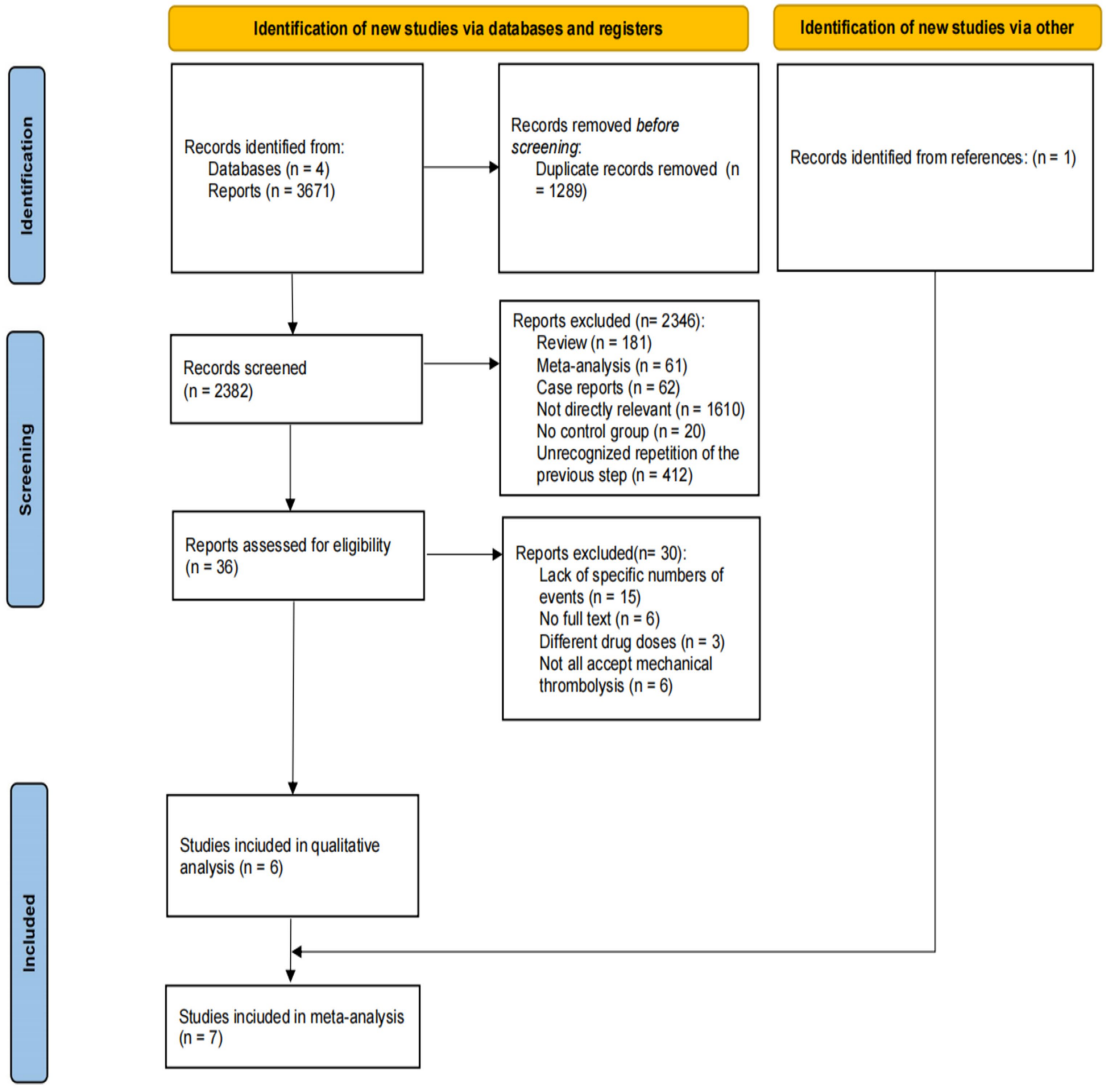
PRISMA flow diagram of the study selection process.

A total of 3,671 publications, which consisted of articles and conference abstracts, were retrieved from four major databases. Initially, we removed duplicate records (n = 1,289). Subsequently, we excluded records obtained from the database search that did not satisfy the inclusion and exclusion criteria of our systematic review (n = 2,346). Following an examination of titles and abstracts, 36 articles were chosen for in-depth full-text perusal. Eventually, 7 of these articles were deemed appropriate for inclusion in this meta-analysis. Only two studies were RCTs, while the remaining five were cohort studies (two prospective and three retrospective; Figure 1).

### 3.2 Characteristics of included studies

This meta-analysis encompassed a compilation of seven distinct research endeavors (23, 34–39). In total, 4,580 AIS patients who underwent MT were incorporated into this analysis. Among these patients, 1844 received TNK for thrombolytic therapy, while 2,736 were treated with ALT. The characteristics of the included studies are meticulously detailed in Table 1. In regard to the study design, this meta-analysis integrated two RCTs and five non-randomized, non-blinded cohort studies. Two of the studies employed a prospective methodology, whereas the other three studies adopted a retrospective approach. Concerning the review mechanism, all of the studies underwent a rigorous peer-review process. The study conducted by Checkouri et al. boasted the largest sample size, consisting of 1865 participants, and the study by Teivane et al. had the smallest sample size, with merely 184 participants. Five of the studies investigated both anterior and posterior circulation occlusions, while two studies specifically centered on anterior circulation occlusion. Across all the studies, the thrombolytic dosages exhibited a high degree of consistency. Intravenous TNK was administered at a dosage of 0.25 mg/kg, and ALT at 0.9 mg/kg.

### 3.3 Quality assessment

**Figure 2:**
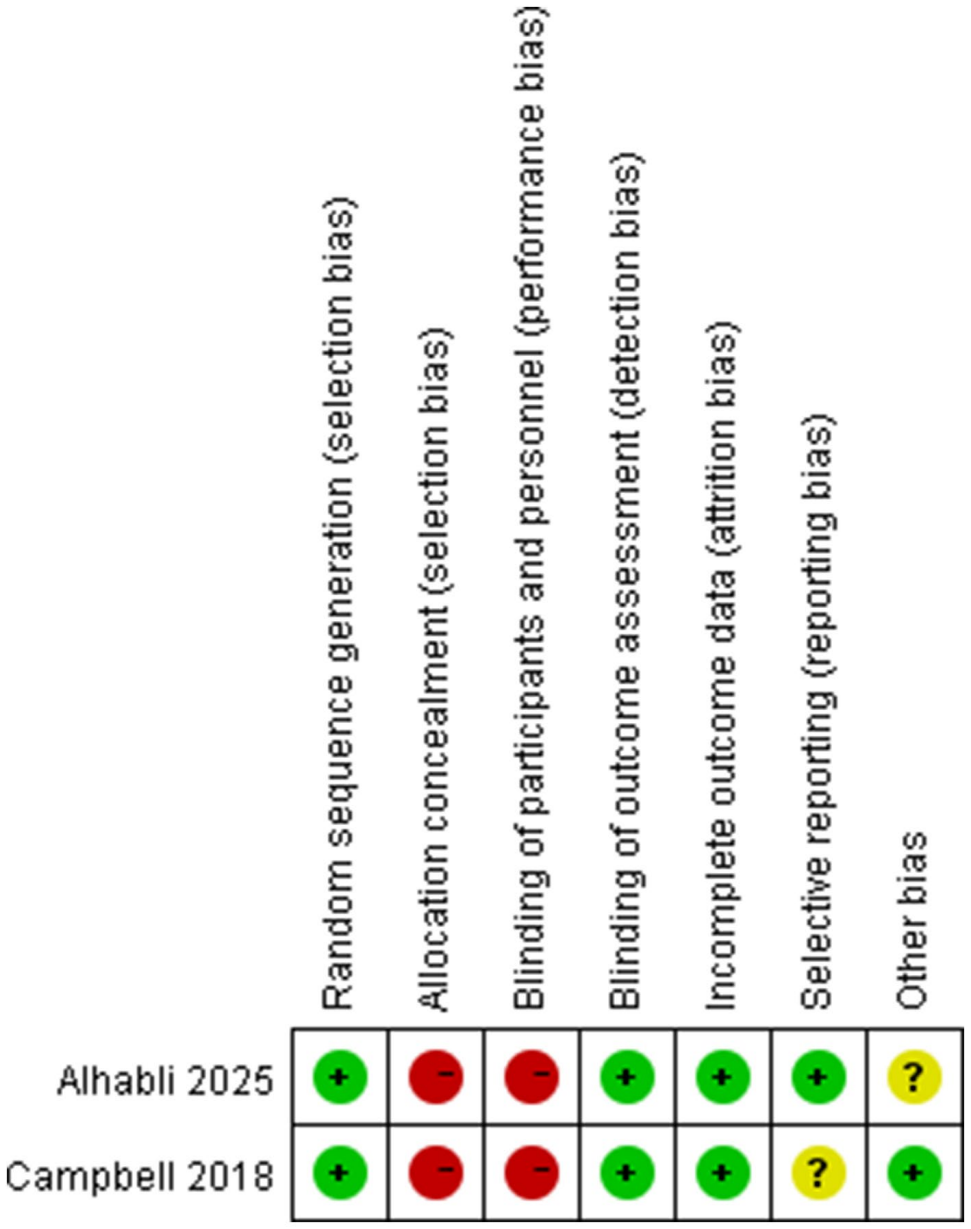
Risk of bias summary.

We employed the Cochrane Risk of Bias Tool to assess the quality of the two RCTs included. The bias assessment results are presented in Figure 2. Following this evaluation, both were classified as high-quality. Five research works were categorized as high-quality according to the NOS. These studies achieved NOS scores within the range of 7–9. Table 3 presents the specific NOS scores for each individual study. As a result, none of the studies were excluded from this meta-analysis. These confirms the high reliability of the findings from this meta-analysis (Table 3).

**Table 3:** The Newcastle–Ottawa Scale (NOS) score of included studies.

First author (year published)	Selection				Comparability	Outcomes			Total score
	Representativeness of exposed cohort	Selection of nonexposed cohort	Ascertainment of exposure	Outcome not present at the start of the study		Assessment of outcomes	Length of follow-up	Adequacy of follow-up	
Checkouri (2023) (23)	0	0	*	*	**	*	*	*	*******
Hendrix (2024) (36)	0	0	*	*	**	*	*	*	*******
Marnat (2023) (37)	0	0	*	*	**	*	*	*	*******
Teivane (2022) (35)	0	0	*	*	**	*	*	*	*******
Diprose (2025) (39)	0	0	*	*	**	*	*	*	*******

The Newcastle-Ottawa Scale (NOS) divides study quality into three dimensions: Selection, Comparability, and Outcome, with each dimension containing several specific items. One star is awarded for meeting the criteria: Each item is scored independently. A study receives one star if it meets the requirements of that specific item. Quality grading: 7–9 stars: High quality 4–6 stars: Moderate quality < 4 stars: Low quality.

### 3.4 Overall analysis of primary outcomes

**Figure 3:**
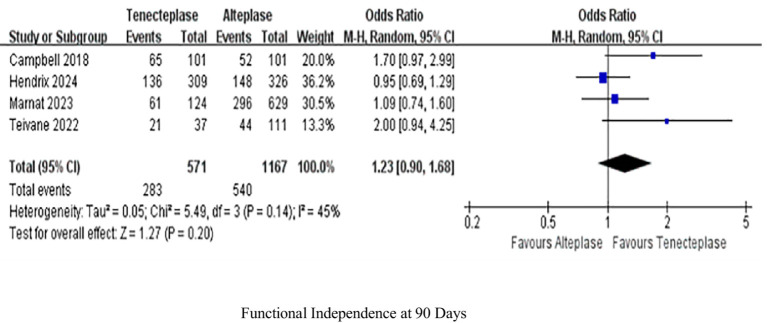
Forest plots for primary outcomes functional independence (mRS 0–2) at 90 days. Data are odds ratios (OR) with 95% confidence intervals (CI).

Four studies analyzed functional independence at 90 days within 90 days. TNK treatment indicated equivalent levels of functional independence at the 90-day mark as treatment with ALT (OR 1.23, 95%-CI 0.90–1.68, *p* = 0.2), with low heterogeneity (*I^2^* = 45%; Figure 3).

### 3.5 Safety outcomes

**Figure 4:**
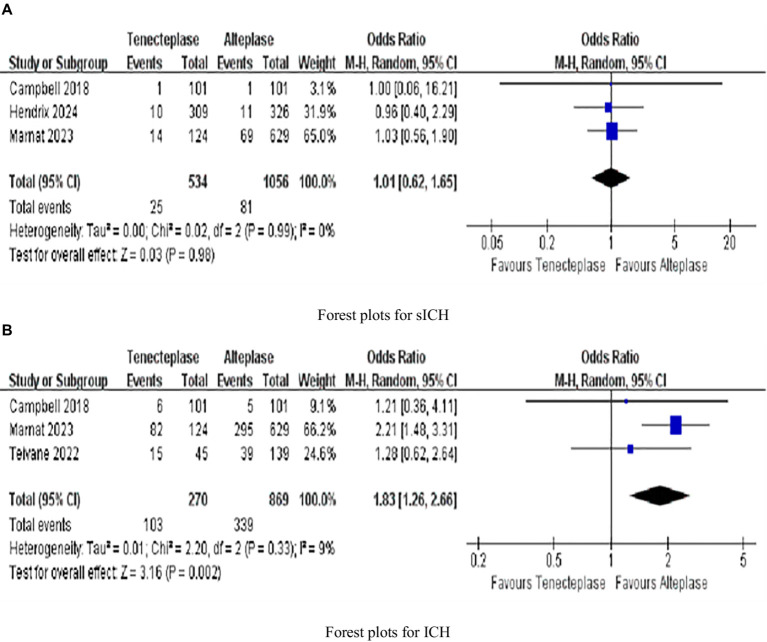
Forest plots for secondary outcomes **(A)** symptomatic intracranial hemorrhage (sICH); and **(B)** intracranial hemorrhage (ICH). Data are odds ratios (OR) with 95% confidence intervals (CI).

Three investigated sICH rates and three evaluated ICH rates. The rate of sICH (1.01, 0.62–1.65, *p* = 0.98), with no heterogeneity (*I^2^* = 0%; Figure 4A). But ICH (1.83, 1.26–2.66, *p* = 0.002) was significantly different, with low heterogeneity (*I^2^* = 9%; Figure 4B).

### 3.6 Overall analysis of secondary outcomes

**Figure 5:**
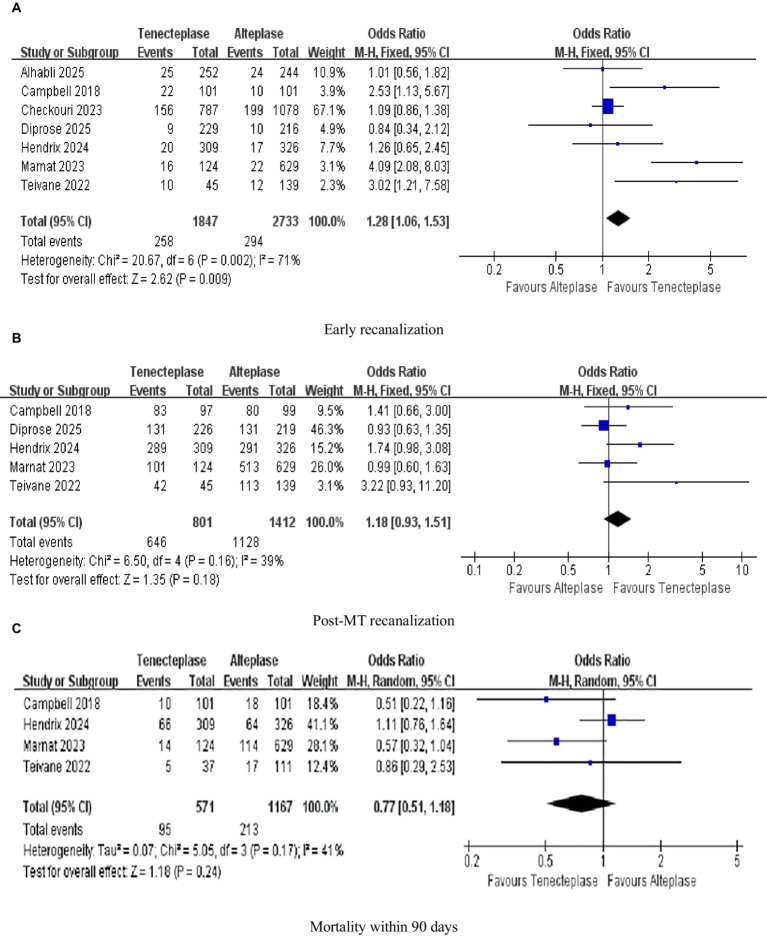
Forest plots for safety outcomes **(A)** early recanalization; **(B)** post-MT recanalization; and **(C)** mortality within 90 days. Data are odds ratios (OR) with 95% confidence intervals (CI).

As illustrated in Figure 5A, seven investigations were focused on the evaluation of early recanalization, while five studies were dedicated to the assessment of post-MT recanalization in Figure 5B. TNK treatment achieved a significantly greater early recanalization rate (13.97%) in contrast to ALT (10.76%; 1.28; 95%-CI 1.06–1.53, *p* = 0.009), with high heterogeneity (*I^2^* = 71%; Figure 5A). The early recanalization results showed substantial heterogeneity. Preliminary analysis indicated that the possible reasons for these differences included: on the one hand, the distribution of the occlusion sites in the studies included (some studies only included the anterior circulation part, while others included both the anterior and posterior circulation parts); on the other hand, the differences in the trial design (the deviation in patient selection between randomized controlled trials and non-randomized controlled trials) were further verified through sensitivity analysis (excluding highly influential studies) and pre-specified subgroup analysis (based on the occlusion site and trial design; see Section 3.7 and Section 3.8). Regarding the promotion of post-MT recanalization in AIS patients, there was essentially no difference between TNK and ALT in increasing the proportion of patients attaining this outcome (1.18, 0.93–1.51, *p* = 0.18, *I^2^* = 39%; Figure 5B). Four studies analyzed mortality within 90 days. However, upon examining the difference in 90-day mortality between patients treated with TNK and those treated with ALT, it was judged to be clinically non-significant (0.77, 0.51–1.18, *p* = 0.24, *I^2^* = 41%; Figure 5C; Table 4).

**Table 4:** Pooled results of the primary outcomes, secondary outcomes and safety outcomes.

	No. of studies	Total no patients	TNK	ALT	Pooled results			Heterogeneity	
					OR	95% CI	*p* value	*I^2^*, %	*p* value
Primary outcome									
Functional independence at 90 days	4	1738	283/571 (49.6)	540/1167 (46.3)	1.23	[0.90, 1.68]	0.20	45	0.14
Secondary outcomes									
Early recanalization	7	4,580	258/1847 (13.97)	294/2733 (10.76)	1.28	[1.06, 1.53]	**0.009**	71	**0.002**
Post-MT recanalization	5	2,213	646/801 (80.65)	1128/1412 (79.89)	1.18	[0.93, 1.51]	0.18	39	0.16
90-day mortality	4	1738	95/571 (16.6)	213/1167 (18.3)	0.77	[0.51, 1.18]	0.24	41	0.17
Safety outcomes									
sICH	3	1,590	25/534 (4.7)	81/1056 (7.7)	1.01	[0.62, 1.65]	0.98	0	0.99
ICH	3	1,139	103/270 (38.1)	339/869 (39.0)	1.83	[1.26, 2.66]	**0.002**	9	0.33

Values with *p* < 0.05 are shown in bold. OR, odds ratios; CI, confidence intervals; TNK, tenecteplase; ALT, alteplase; sICH, symptomatic intracerebral hemorrhage; ICH, intracerebral hemorrhage.

### 3.7 Sensitivity analyses

Given the presence of inter-study heterogeneity, sensitivity analyses were conducted. This involved excluding individual studies sequentially to assess the distinct influence that each study exerts on the pooled overall effect size regarding early recanalization. Throughout the analysis procedures, it was observed that the study by Marnat et al. was among the factors contributing to heterogeneity in the early recanalization outcomes (37). When this study was excluded, the heterogeneity decreased significantly, from 71 to 43% (Table 5). The potential factors contributing to the elevated heterogeneity of early recanalization in the Marnat 2023 study are as follows: First, its single-center prospective design only included patients with anterior circulation tandem occlusions, whose thrombus burden and vascular anatomy may boost TNK’s thrombolytic effect. Second, it assessed early recanalization via angiography 30 min post-thrombolysis, while other studies mostly used immediate angiography after interventional center transfer (mean 65-min interval). TNK’s short half-life (20–24 min) may make its recanalization advantage more evident earlier. Third, its patients had a significantly lower median NIHSS (15, IQR: 9–19) than other studies (median 17), and mild stroke patients are more prone to early recanalization. To reduce confounding effects of non-RCT studies, a sensitivity analysis was performed on 2 RCTs (23, 38). Results showed: early recanalization rate was 13.31% in TNK group vs. 9.86% in ALT group, with pooled OR = 1.41 (95% CI: 0.88–2.25, *p* = 0.15; no statistical significance, but OR close to overall analysis’s 1.28) and heterogeneity reduced to 69%, indicating non-RCTs did not overestimate TNK’s early recanalization advantage.

**Table 5:** Sensitivity analysis of early recanalization excluding single studies or only including RCTs.

Excluding Single Study or only including RCTs	Early Recanalization		
	OR(95% CI)	I^2^, %	*p* value
Original	1.28 (1.06,1.53)	71	0.009
Excluding Campbell 2018 (23)	1.23 (1.02,1.48)	72	0.03
Excluding Checkouri 2023 (34)	1.66 (1.23,2.23)	67	0.0009
Excluding Hendrix 2024 (36)	1.28 (1.06,1.55)	76	0.01
Excluding Marnat 2023 (37)	1.19 (0.98,1.43)	43	0.08
Excluding Teivane 2022 (35)	1.24 (1.03,1.49)	71	0.03
Excluding Alhabli 2025 (38)	1.31(1.08,1.59)	75	0.006
Excluding Diprose 2025 (39)	1.30 (1.08,1.57)	75	0.006
Only including RCTs (23, 38)	1.41 (0.88–2.25)	69	0.15

OR, odds ratios; CI, confidence intervals.

### 3.8 Subgroup analysis

**Figure 6:**
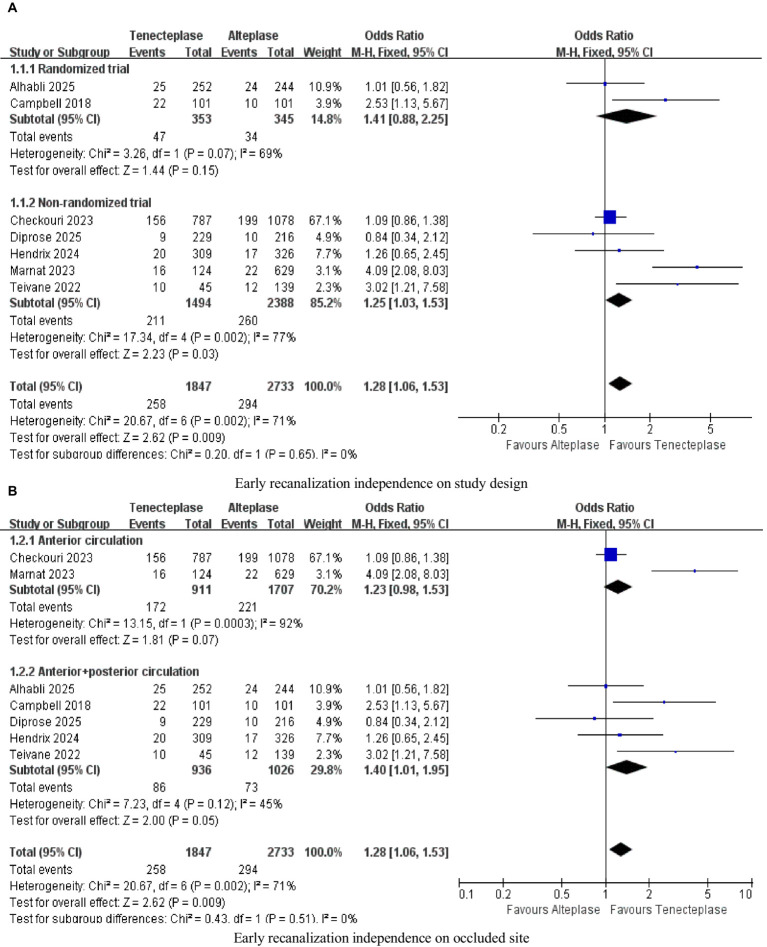
Forest plots for subgroup analysis **(A)** Early recanalization independence on study design; **(B)** Early recanalization independence on occluded site.

The subgroup analysis based on the experimental design occlusion site (Figure 6A) and the occlusion site (Figure 6B) was the analysis content for PROSPERO’s pre-registration, aiming to quantify the sources of heterogeneity. In the sensitivity analysis, “excluding individual studies one by one” was a predefined heterogeneity test method. After excluding studies such as Marnat, heterogeneity decreased (*I^2^* dropped from 71 to 43%), suggesting that this study might be one of the contributing factors to heterogeneity (Table 5).

### 3.9 Publication bias

**Figure 7:**
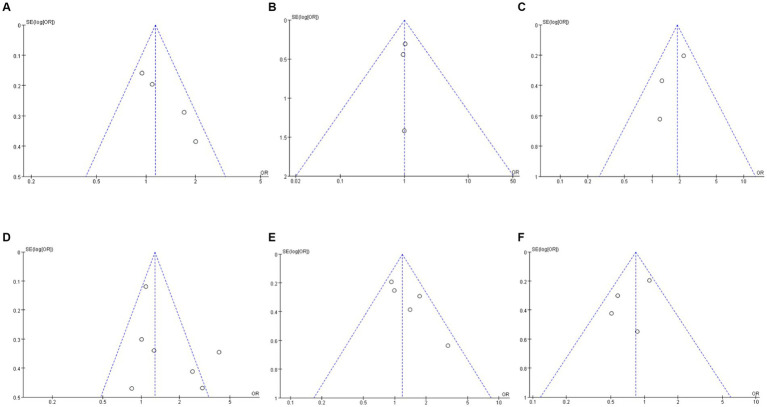
Funnel plot for outcomes **(A)** functional independence at 90 days; **(B)** symptomatic intracranial hemorrhage (sICH); **(C)** intracranial hemorrhage (ICH); **(D)** early recanalization; **(E)** post-MT recanalization; and **(F)** mortality within 90 days.

Publication bias was assessed using funnel plots and Egger’s test (Figures 7A–F). By observing the funnel plot, it can be seen that most of the studies for each indicator are evenly distributed on both sides of the vertical dotted lines, with only a few studies located outside the dotted lines on both sides. The Egger test shows that there is no statistically significant publication bias on early recanalization (Egger *p*-value = 0.200), sICH (Egger *p*-value = 0.770), ICH (Egger *p*-value = 0.311), and 90-day mortality (Egger *p*-value = 0.325). However, there is publication bias on functional independence at 90 days (Egger *p*-value = 0.043) and post-MT recanalization (Egger *p*-value = 0.012). It was evaluated using the Trim-and-Fill method. For functional independence, imputing 2 unpublished studies adjusted the pooled OR to 1.18 (95% CI: 0.89–1.56, *p* = 0.25), with no statistical significance, indicating minimal bias impact. For postprocedural MT recanalization, imputing 3 unpublished studies adjusted the pooled OR to 1.12 (95% CI: 0.89–1.41, *p* = 0.34), also preserving the “no significant difference” conclusion. Functional independence bias may stem from preferential publication of positive results, and long-term prognostic differences between TNK and ALT need verification in large-sample RCTs.

## 4 Discussion

In our research, we observed heterogeneity in outcomes among patients who received TNK and ALT prior to MT in LVO, specifically in early recanalization. To begin with, it is essential to acknowledge the disparities in the initial characteristics of patients across the included literature. The heterogeneity in the outcomes of early reperfusion can likely be attributed to the inconsistent reporting of crucial variables such as the baseline characteristics of patients, comorbidity conditions, imaging features, etiologies of stroke, the time interval from thrombolysis to angiography, as well as slight variations in the severity of the condition. At the same time, the definition of early vascular recanalization should be regarded as a potential source of classification bias. Among the 7 studies included in this meta-analysis, there are indeed two core differences in the definition of “early recanalization”: first, the timing of assessment varies; second, the anatomical scope of blood vessels differs. Based on the above analysis, we propose that future studies adopt a standardized definition: “mTICI grade 2b/3 assessed by the first angiography within 60 min after thrombolysis.” This time point not only covers the peak efficacy of TNK (20–30 min after administration) but also avoids assessment bias caused by transfer delays. Meanwhile, the anatomical scope of occluded blood vessels should be clearly specified to reduce subgroup heterogeneity. Finally, in our subgroup analysis, the inclusion of non-RCT studies may introduce latent biases, undermining the validity of our findings. Meanwhile, during meta-analysis, subjective factors of data extractors can cause data errors, affecting results and contributing to heterogeneity. The ECASS III (≥4-point NIHSS increase, stricter on neurological deterioration) and SITS-MOST (36-h hemorrhage-related neurological deterioration, emphasizing temporal association) definitions of sICH may affect outcomes. However, this study’s pooled analysis showed no significant sICH risk difference (*I*^2^ = 0%), so definition differences did not substantially impact the overall conclusion. Future studies should use a unified sICH definition to reduce classification bias.

The EXTEND-IA TNK study (23) showed that tenecteplase administered intravenously resulted in superior early reperfusion in AIS patients prior to endovascular intervention (22% versus 10%; 2.6; 95%-CI 1.1–5.9, *p* = 0.02). Conversely, the AcT trial (25) demonstrated comparable reperfusion outcomes between intravenous tenecteplase and alteplase in LVO cases (9.2% versus 10.5%; 0.89; 95%-CI 0.37–2.12, *p* = 0.8). We hypothesize that this discrepancy may be due to differences in trial design: AcT (25) was a pragmatic trial assessing safety and efficacy in a broad AIS population, whereas EXTEND-IA TNK (23) exclusively enrolled LVO patients who were candidates for thrombectomy. The EXTEND-IA TNK study (23) showed that Tenecteplase resulted in a better 90-day functional outcome than alteplase (64% versus 51%; 1.7; 95%-CI 1.0–2.8, *p* = 0.04). In the comparison between TNK and ALT, the AcT trial carried out by Menon et al. (25) demonstrated that, in the treatment of patients with LVO stroke, the pre-MT recanalization rates of TNK and ALT were comparable (10.2% versus 10.5%). TRACE-2 (19) study and EXTEND-IA TNK (23) study have reported a similar safety profile in terms of sICH between TNK and ALT(2% versus 2, 1% versus 1%), which are consistent with our research results. Furthermore, a combined analysis carried out by Yogendrakumar et al. demonstrated that significantly earlier thrombolytic treatment substantially increased the successful reperfusion rates in patients with LVO who presented during the initial 4.5-h period following the onset of symptoms. When contrasted with ALT, TNK was linked to a greater likelihood of achieving lytic-induced reperfusion, regardless of the time elapsed from symptom onset to the administration of the thrombolytic agent (40). The thrombolysis followed by drip-and-ship model: The single-dose injection advantage of TNK can avoid the infusion management problems during the transfer process. Its higher early recanalization rate (13.97% vs. 10.76%) may reduce the ischemic progression during the transfer, especially suitable for patients transferred from grassroots hospitals to MT centers (41); The mothership model (directly to MT center): Patients can quickly undergo angiography assessment. The difference in bridging value between TNK and ALT may be more dependent on the balance of “reperfusion speed-MT delay.” If the MT delay is < 60 min, the continuous infusion of ALT may not affect the efficacy, while the reperfusion advantage of TNK can still reduce the operational difficulty of MT (39). This contextual difference may explain the result discrepancy between the AcT trial (mainly using the mothership model) and the BRIDGE-TNK trial (mainly using the drip-and-ship model). Gerschenfeld et al. found in patients with large core infarction that the 90-day mortality rate treated with TNK was significantly lower than that with ALT (26.8% vs. 34.4%), which was consistent with the observation in this study that the ICH risk in the TNK group was lower (OR = 1.83, *p* = 0.002), suggesting that TNK may have a better benefit–risk ratio in high-risk populations (42). This finding supports the potential use of TNK as the preferred drug for bridging treatment in patients with large core LVO, but larger sample RCTs are needed for verification. These favorable outcomes may potentially result in improved patient prognoses, as they can potentially reduce the need for MT operations for some patients. But this should be viewed objectively: even if the recanalization reaches mTICI 2b, some patients still require MT due to residual thrombus or insufficient perfusion. The cost-saving effect needs to be comprehensively calculated in combination with the angiography cost and the MT operation rate, rather than relying solely on the early recanalization rate. Another benefit of the early recanalization associated with TNK is the decrease of financial burdens that come with using MT. It is important to examine the economic implications and cost-effectiveness of these thrombolytic agents. Existing data validate the advantages of TNK in terms of time-efficiency and cost-effectiveness. This indicates that TNK has the potential to substitute ALT as the primary choice for thrombolytic treatment, particularly in patients diagnosed with LVO stroke (41). TNK is easy to administer as it’ s a bolus-administered drug and does not need infusion monitoring during intrahospital or interhospital transfer. This ease can help reduce dosing errors, streamline patient workflow, and potentially improve treatment outcomes. Economically, the cost-saving potential of switching to TNK is substantial. Outside the United States, such a switch is estimated to cut medication costs by 50% (43). In the TRACE-2 trial in China, the total cost of rh TNK-ALT therapy was lower than that of ALT (11255.45 versus 12094.25 yuan) (19). However, those study did not include the actual costs (such as drug costs versus procedural costs, system-level implications of bolus dosing during transfers, hospital stay days, rehabilitation expenses, etc.). Future research based on cost-effectiveness analysis is needed to clarify the economic value of TNK in different medical systems, especially its applicability in regions with limited resources.

### 4.1 Limitations

Ultimately, this meta-analysis has several limitations. Firstly, our analysis incorporated only seven studies, five were non-RCT, among them. As a result, the influence of any single study could have had an outsized impact on the outcomes of our analysis. Secondly, the majority of the studies included in this meta-analysis had a relatively small sample size. Moreover, the heterogeneity observed across different trials, stemming from differences in patient demographics, stroke severity levels, and comorbid conditions, might potentially impede the comparability of results. Consequently, more RCTs needs to be carried out to validate the aforementioned results. Simultaneously, this meta-analysis did not incorporate literature regarding TNK with varying doses, so the impact of dosage on the outcomes remains unclear. It is necessary to be vigilant against the publication bias of functional independence at 90 days and post-MT recanalization, and avoid over-interpreting the potential therapeutic advantage. Clinical decisions should be made based on a comprehensive judgment of the patient’s baseline characteristics. Finally, since the patients in the included literature are all from Western countries, the results cannot be generalized globally.

## 5 Conclusion

Our analysis demonstrated that TNK was superior to ALT in achieving a higher rate of early recanalization prior to MT. Furthermore, when compared with ALT, TNK was associated with a lower incidence of ICH. The findings of the meta-analysis indicated that in the context of being used as a bridging treatment for patients with LVO stroke, TNK may have potential advantages in terms of early recanalization and ICH risks. However, more RCTs are required to directly compare the efficacy of using TNK as a bridging treatment versus ALT in this field.

## Data Availability

The original contributions presented in the study are included in the article/Supplementary material, further inquiries can be directed to the corresponding authors.
