# Supplementary material for: Tenecteplase versus alteplase in bridging therapy in patients with large vessel occlusion stroke: a meta-analysis
Source: Front Neurol. 2025 Nov 5;16:1661357. doi: 10.3389/fneur.2025.1661357 (PMC12626840; doi:10.3389/fneur.2025.1661357)
Supplement: Supplementary file 1 [file Data_Sheet_1.pdf]

Tenecteplase versus alteplase In bridging therapy in Patients with Large Vessel Occlusion Stroke : a meta-analysiss

Supplementary Materials

Supplemental Table 1. Search strategy for PubMed
Supplemental Table 2. Search strategy for Embase
Supplemental Table 3. Search strategy for Web of Science
Supplemental Table 4. Search strategy for Cochrane Library

Supplemental Table 1. Search strategy for each database

PubMed search		387 articles
No.	Search term	
1	(Alteplase [Title/Abstract]) OR (Tenecteplase [Title/Abstract])	
2	((((Ischemic Stroke [Title/Abstract]) OR (Ischaemic Stroke [Title/Abstract])) OR (Cerebral Infarction [Title/Abstract])) OR (Middle Cerebral Artery Infarction [Title/Abstract])) OR (Middle Cerebral Artery Stroke [Title/Abstract])))	
3	Thrombectomy [Title/Abstract]	
4	1 AND 2 AND 3	

Supplemental Table 2. Search strategy for Embase

Embase search		1632 articles
No.	Search term	
1	'alteplase'/exp	
2	'tenecteplase':ab,ti	
3	'ischemic stroke':ab,ti	
4	'ischaemic stroke':ab,ti	
5	'cerebral infarction':ab,ti	

6	'middle cerebral artery infarction':ab,ti
7	'middle cerebral artery stroke':ab,ti
8	'thrombectomy':ab,ti
9	1 OR 2
10	3 OR 4 OR 5 OR 6 OR 7
11	8 AND 9 AND 10

Supplemental Table 3. Search strategy for Web of Science

Web of Science search	1472 articles
No.	Search term
1	('alteplase' OR ' tenecteplase')
2	('ischemic stroke' OR 'ischaemic stroke' OR 'cerebral infarction' OR 'middle cerebral artery infarction' OR ' middle cerebral artery stroke ')
3	'thrombectomy'
4	1 AND 2 AND 3

Supplemental Table 4. Search strategy for Cochrane Library

Cochrane Library	130 articles
No.	Search term
1	MeSH descriptor: [Tissue Plasminogen Activator] explode all trees
2	('Tenecteplase'):ti,ab,kw
3	MeSH descriptor: [Ischemic Stroke] explode all trees
4	('Ischaemic stroke'):ti,ab,kw OR ('Cerebral infarction'):ti,ab,kw OR ('Middle cerebral artery infarction'):ti,ab,kw OR ('Middle cerebral artery stroke'):ti,ab,kw
5	MeSH descriptor: [Thrombectomy] explode all trees
6	1 OR 2
7	3 OR 4
8	5 AND #6 AND #7
